# Indoor Motion Detection Using Wi-Fi Channel State Information in Flat Floor Environments Versus in Staircase Environments

**DOI:** 10.3390/s18072177

**Published:** 2018-07-06

**Authors:** Zehua Dong, Fangmin Li, Julang Ying, Kaveh Pahlavan

**Affiliations:** 1 School of Information Engineering, Wuhan University of Technology, Wuhan 430070, China; zdong2@wpi.edu; 2 Department of Mathematics and Computer Science, Changsha University, Changsha 410083, China; 3 Department of Electrical and Computer Engineering, Worcester Polytechnic Institute, Worcester, MA 01609, USA; jying@wpi.edu (J.Y.); kaveh@wpi.edu (K.P.)

**Keywords:** motion detection, Wi-Fi, CSI, indoor, staircase, fall detection, intruder detection

## Abstract

Recently, Wi-Fi channel state information (CSI) motion detection systems have been widely researched for applications in human health care and security in flat floor environments. However, these systems disregard the indoor context, which is often complex and consists of unique features, such as staircases. Motion detection on a staircase is also meaningful and important for various applications, such as fall detection and intruder detection. In this paper, we present the difference in CSI motion detection in flat floor and staircase environments through analysing the radio propagation model and experiments in real settings. For comparison in the two environments, an indoor CSI motion detection system is proposed with several novel methods including correlation-based fusion, moving variance segmentation (MVS), Doppler spread spectrum to improve the system performance, and a correlation check to reduce the implementation cost. Compared with existing systems, our system is validated to have a better performance in both flat floor and staircase environments, and further utilized to verify the superior CSI motion detection performance in staircase environments versus flat floor environments.

## 1. Introduction

With the development of electronic and computer technologies, motion detection using various signals has been researched and efficiently utilized in health care [1,2], entertainment and social security, thus attracting increasing academic and industrial attention. Compared with conventional motion detection systems using sensors [3], vision and radar, channel state information (CSI)-based systems using Wi-Fi signals have the advantage of being device-free, requiring no additional or specific devices to provide passive service. For indoor settings such as homes, hospitals and office buildings, the wireless communication service is already established, and additional devices are not necessary. Therefore, CSI-based systems can be implemented at a low cost.

With the rapid commercialization of Wi-Fi devices, CSI-based systems have been generalized to various applications [4], including fall detection [5,6], gesture detection [7], mouth movement detection [8], gait detection [9], human identification [10,11], intruder detection [12] and even indoor localization [13,14], but these systems are mainly designed for flat floor environments and disregard complex indoor settings with features such as staircases, they have not been validated in staircase environments and compared in both flat floor and staircase environments. Staircases are common and important in various types of buildings, including hospitals, homes and offices. Accordingly, CSI applications in staircase environments are also meaningful and can potentially enhance human health care, security and convenience. For example, in the health care setting, patients, particularly elderly patients, are more likely to have a fall incident on a staircase rather than on a flat floor. Therefore, fall detection on a staircase is more important than on a flat floor at home or in a hospital. Moreover, walking detection on staircases can be leveraged to identify which direction a person is walking (e.g., upstairs or downstairs), which can be utilized to automatically control the power supply of lighting fixtures on different floors in smart buildings. This work analyses the difference of CSI motion detection in the two environments through a theoretical radio propagation analysis and experiments in real settings.

**Figure 1:**
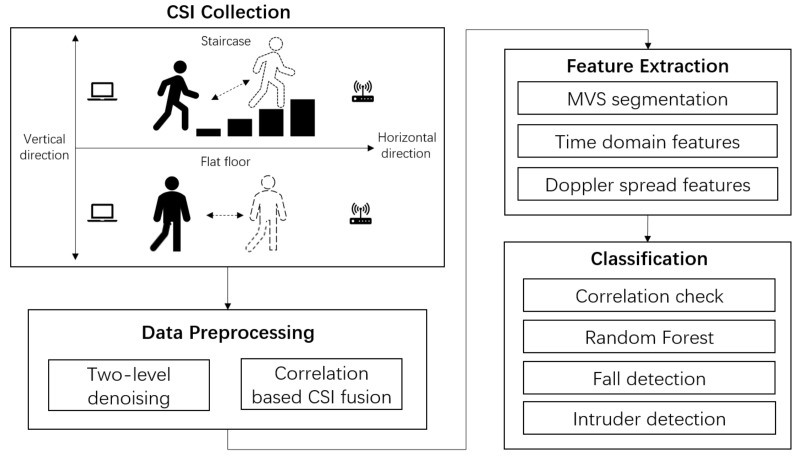
System architecture and data flow.

Compared with a flat floor, when a human is moving on a staircase, he or she moves not only in the horizontal direction but also in the vertical direction, and the legs and arms bend with greater magnitude, as shown in Figure 1. Therefore, radio multi-paths and shadow fading effects in a staircase environment become greater in magnitude and more complicated. Accordingly, wireless signal variances, including received signal strength (RSS) and phase variances generated by motion on a staircase, become larger. Compared to the conventional wireless signal, CSI is a fine-grained wireless signal [13,14,15] containing multiple streams and subcarriers of information to reflect the movement of detected objects. Therefore, the increased wireless signal variance caused by motion on a staircase can be precisely recorded via CSI to achieve better motion detection performance. For instance, CSI-based fall detection and intruder detection or alarm applications can be more sensitive on a staircase than on a flat floor in terms of human safety, health, and security.

In this work, we propose an indoor CSI motion detection system that can be used in both flat floor and staircase settings. The system architecture is illustrated in Figure 1, which includes several novel methods such as correlation-based CSI fusion, moving variance segmentation (MVS), Doppler spread features and the correlation check to improve system performance.

The main contributions of our work are as follows:

- We present the difference in CSI motion detection in the two environments through a theoretical radio propagation model and experiments in real settings. The difference is analysed from the perspective of signal variance magnitude caused by the human movement direction.
- For comparison in the two environments, we propose a new system with several novel methods including the correlation-based fusion, MVS segmentation and Doppler spread spectrum to improve the system performance, and a correlation check to reduce the implementation cost. The proposed system is first validated to have a better performance compared to existing systems, and further utilized to verify the superior performance of CSI motion detection in staircase environments versus flat floor environments.
- Experiments in a real setting also validate the efficiency of the random forest (RF) algorithm for CSI motion detection in the two environments compared with that of the support vector machine (SVM) and K-nearest neighbour (KNN) algorithms.

The rest of the article is organized as follows. Related literature on motion detection systems will be investigated in Section 2. We introduce basic preliminary variables, including differences in motion detection scenarios in two environments, a brief background of CSI, and the energy distribution of frequency components in Section 3. Then, we describe the methodologies of our proposed system in Section 4. Most importantly, the system performances are reported and discussed in Section 5. In Section 6, the overall work is summarized and our conclusions are presented.

## 2. Related Work

Motion detection systems can be classified into four categories according to the technologies applied: sensor-, vision-, radar-, and CSI-based systems. Traditionally, sensors [16,17,18,19] are the most common equipment used due to the convincing classification rate and simplified practical implementation. A wearable self-powered sensor was proposed in [17] to detect human finger motions for human computer interfacing and osteoarthritis rehabilitation applications. Geng et al. laid the foundation of wearable sensor based motion detection using radio frequency (RF) signals and proposed a fire-fighter motion monitoring system for first responders [3]. Moreover, motion detection using the low-cost Microsoft Kinect sensor (Microsoft Corporation, Redmond, WA, USA) has also been widely researched [18]. A fall detection system that measures velocity based on contraction or expansion of the width, height and depth of a 3D bounding box was proposed in [19]. Computer vision-based motion detection using image processing has been adopted since the 2000s [20]. An analysis of comparative surveys of gesture detection based on vision was provided in [21], where hand gestures as a natural interface motivated research on gesture detection and representation technologies based on vision. Han et al. [2] proposed a vision-based unsafe action detection system for behavior monitoring to detect unsafe actions during ladder climbing. In addition, various types of radar [22,23,24] also have the potential for motion detection. Jokanovic et al. considered radar for fall detection using time-frequency analysis based on a deep learning approach [25]. A system for detecting human respiratory signals through a wall using ultra-wide-band (UWB) radar was proposed in [26], with potential applications in battlefields and victim searches. Yi et al. [27] propose a device-free sensing technology for personnel detection in a foliage environment using the UWB transceiver. Because these approaches require specific equipment and pre-installation requirements, sometimes they are limited by critical cases.

Compared to traditional systems requiring specific hardware, a CSI-based system enables passive and non-invasive detection using existing commercial Wi-Fi devices without additional equipment requirements. WiFall [5] proposed device-free fall detection using CSI by analyzing correlations between CSI amplitude variations and human motions. RT-Fall exploits both CSI amplitude and phase to detect falls automatically [6]. WiGest leverages the received signal strength indicator (RSSI) of CSI to detect hand motions [7]. CARM [28] is an activity recognition system based on two theoretical models: CSI-speed and CSI-activity models. WiHear enables CSI to “hear” humans talk through mouth motion profiles, which leverage partial multipath effects and wavelet packet transformation. In addition to motion detection, CSI-based systems have also been introduced to realize human identification. Zimu et al. [29] propose the concept of omnidirectional passive human detection using CSI features. WifiU identifies humans by capturing human gait patterns based on a spectrogram generated from CSI. To best characterize walking patterns, WifiU performs autocorrelation on the torso reflection to remove imperfections in spectrograms [9]. Based on accurate gait analysis through noise estimation, Chen et al. adaptively fused CSI and adopted acoustic measurements to achieve robust person identification [10]. WiWho also identifies a human from a small group of people using CSI gait patterns and demonstrates how step and walk analysis can be used to identify personal gait [30]. CareFi [12] extracts CSI to analyze distinguishing gait characteristics for intruder sensing. Although CSI-based systems have been researched for various applications, they are mainly suitable for flat floor environments; no system has been specified for staircase environments.

## 3. Preliminaries

### 3.1. Differences in Motion Detection Scenarios in Flat Floor and Staircase Environments

Wireless signals can be leveraged to detect human motion mainly based on the signal variance caused by radio multi-path and shadow fading effects. According to the Friis free space Equation (1) [31], the received signal variance is affected by the distance from the signal transmitter to receiver *d*, $P_{t}$ is the transmitted power, $G_{r}$ and $G_{t}$ are the receiver and transmitter antenna gains, respectively, and λ is the radio wavelength in meters:

(1) $$\frac{P_{r}}{P_{t}}=G_{r}\times G_{t}\times {\left(\frac{\lambda }{4\pi d}\right)}^{2}.$$

When humans move in an indoor environment, the characteristics of wireless channels from multi-paths fluctuate over time and can be recorded as amplitude and phase variances. The multi-paths are categorized into line-of-sight (LOS) paths and reflected non-line-of-sight (NLOS) paths based on the surroundings, such as the ceiling, the floor, walls, furniture and moving humans. The total received power of receiver $P_{a}$ is composed of the static power $P_{s}$ generated by static surroundings including the floor, the ceiling and furniture; the dynamic power $P_{d}$ is caused by dynamic surroundings, such as moving humans. Because the static surroundings are always unchanging, fluctuation of the total received power $\Delta P_{a}$ is mainly caused by dynamic human motion and can be analyzed using Equation (2) [5]. $d_{1}$ and $d_{2}$ are the distances between the human and the Wi-Fi transmitter and receiver in the horizontal direction, respectively. $h_{i}$ represents the height of human body scattered points in the vertical direction, *h* is the maximum range of height variance, and $\Delta P_{d}$ is the dynamic power change caused by scattered human body points. As shown in Equation (2), $\Delta P_{d}$ is dominated by the distances $d_{1}$ and $d_{2}$ and height $h_{i}$:

(2) $$\begin{array}{cc} \Delta P_{d}\left(d_{1},d_{2},h_{i}\right) & =\sum_{h_{i}}{\frac{P_{t}G_{t}G_{r}\lambda^{2}}{{\left(4\pi \right)}^{3}\left(d_{1}^{2}+h_{i}^{2}\right)\left(d_{2}^{2}+h_{i}^{2}\right)}}^{},\\ h_{i} & \in (0,h]. \end{array}$$

**Figure 2:**
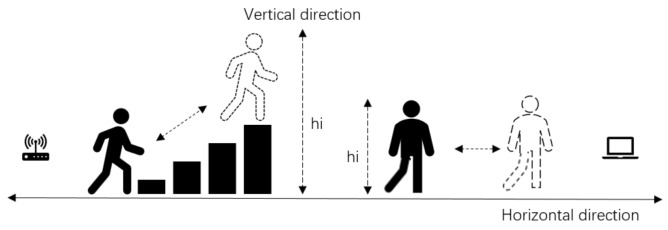
Different motion detection scenarios in the two environments.

In flat floor environments, a human moves only in the horizontal direction and the existing CSI motion detection systems mainly leverage $\Delta P_{d}$, which is dominated by the horizontal variances of $d_{1}$ and $d_{2}$. Compared with flat floor environments, a human moves in both the horizontal and vertical directions in staircase environments as shown in Figure 2. Therefore, not only $d_{1}$ and $d_{2}$ but also vertical variance $h_{i}$ change substantially when a human is moving on a staircase. As illustrated in Equation (2), the variance $h_{i}$ amplifies $\Delta P_{d}$; a larger variance range of $h_{i}$ corresponds to a larger variance range of $\Delta P_{d}$. Therefore, $\Delta P_{d}$ becomes much larger on a staircase than on a flat floor due to the huge variance of $h_{i}$ caused by human movement on a staircase. Because the wireless signal is leveraged to detect human motion mainly using signal fluctuation, the larger $\Delta P_{d}$ can be potentially utilized to achieve better motion detection performance. We do not simply quantify the received power change but analyze the variation based on the model to describe the differences.

### 3.2. Channel State Information

Unlike common received signal strength indication (RSSI), CSI is a fine-grained signal containing streams and subcarrier information. CSI is based on orthogonal frequency-division multiplexing (OFDM) and multiple-input multiple-output (MIMO) technologies and records the wireless signal amplitude and phase information for each pair of transmit-receiver antennas in OFDM subcarriers. As in wireless protocol 802.11n, a 2.4-GHz band can be considered a narrowband flat-fading channel and can be modeled as shown by

(3) $$\vec{Y}=H\vec{X}+\vec{N},$$

where $\vec{X}$ and $\vec{Y}$ represent vectors of the transmitter and receiver, respectively; $\vec{N}$ is a vector of Gaussian noise; and H is the channel matrix and can be estimated as CSI [32], as shown in

(4) $$CSI_{i,j}=\left|CSI_{i,j}\right|exp\left\{j∠CSI_{i,j}\right\},$$

where $\left|CSI_{i,j}\right|$ and $∠CSI_{i,j}$ are the amplitude and phase of subcarrier *j* in stream *i*, respectively. In our work, a Wi-Fi router with two antennas served as the CSI transmitter, and a ThinkPad Laptop (Lenovo Inc., Morrisville, NC, USA) with three antennas equipped with an Intel (Santa Clara, CA, USA) Wi-Fi 5300 (Network Interface Card) NIC served as the CSI receiver. Therefore, we have a 2×3 MIMO wireless communication system, and the CSI data consist of six streams according to the six pairs of transmitter-receiver links. Because the Intel 5300 NIC reports CSI for 30 subcarriers for each stream, which are spread evenly among the 56 subcarriers of a 20-MHz channel or 114 carriers of a 40-MHz channel [33] 6×30=180 data groups are derived from each received CSI packet, as illustrated in Equation (5), where *i* and *j* are the numbers of CSI streams and subcarriers, respectively:

(5) $$CSI_{i,j}=\left[\begin{array}{ccc} CSI_{1,1} & \cdots & CSI_{1,30}\\ \cdots & \ddots & \cdots \\ CSI_{6,1} & \cdots & CSI_{6,30} \end{array}\right].$$

### 3.3. Energy Distribution on Frequency Components

**Figure 3:**
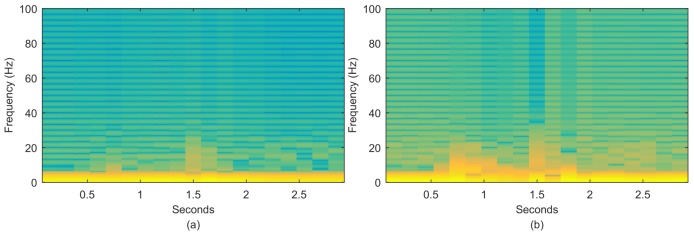
Energy distribution on frequency components: (**a**) walking; (**b**) running.

Similar motions such as walking and running are not easily discriminated based solely on time domain features because the corresponding scattered human body points are similar. However, the energy distribution of their frequency components is distinctive and can potentially improve classification performance. For example, walking and running involve similar body movements, but they correspond to distinctive body movement speeds and powers. We apply a short-term Fourier transformation to profile the spectrograms for walking and running motions performed on a staircase in Figure 3; the faster and more powerful running motion results in more energy distributed in high-frequency components than the lower-speed walking motion. Therefore, the frequency features can be utilized in classification to improve system performance. In our work, the distribution of energy on the motion frequency components is determined using the Doppler spread spectrum and is introduced in Section 4.

## 4. Methodologies

### 4.1. Two-Level Denoising

**Figure 4:**
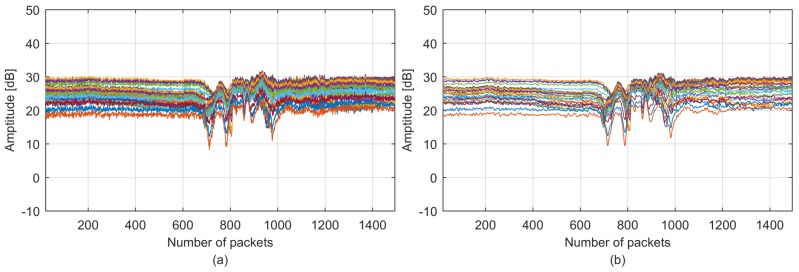
The CSI stream: (**a**) raw stream; (**b**) denoised stream.

The raw CSI stream collected by the Intel 5300 NIC for candidate walking motion is illustrated in Figure 4, which is quite noisy because CSI contains noises generated from indoor surroundings, and the internal state transitions in the wireless signal transmitter and receiver devices are caused by transmission power changes, transmission rate adaptation and internal reference level changes [28]. We should filter noises at the beginning of classification because they can affect classification accuracy and robustness. Corresponding to human motion, the CSI phase can be affected by several factors, such as the carrier frequency offset (CFO) and sampling frequency offset (SFO) [28]. Therefore, we first leverage CSI amplitude variance in this work and explore the CSI phase variance in our future research due to the instability of the phase. We propose a two-level denoising method that applies Hampel and low-pass filters to retain the real CSI stream variance. First, the Hampel identifier [34] is utilized to remove abrupt changes that are clearly not generated by human motions. The Hampel identifier identifies any point falls outside the closed interval [μ−γσ,μ+γσ] as an outlier, where μ and σ are the median and standard deviation of the specified length values, respectively, and γ is an application-dependent parameter. Second, because typical human motion introduces only low-frequency variations [28], we safely remove the high-frequency noises using a low-pass filter. Figure 4(b) illustrates the denoised CSI stream using the two-level denoising method.

### 4.2. Correlation-Based CSI Data Fusion

**Figure 5:**
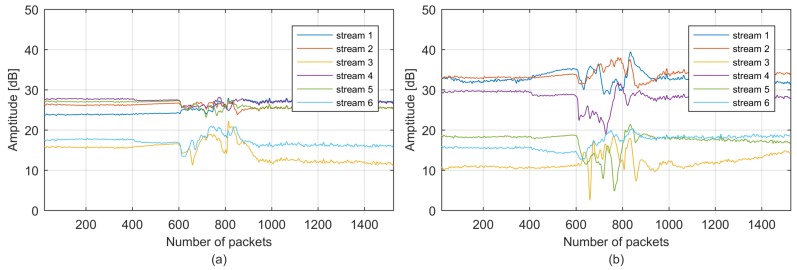
CSI subcarrier fusion: (**a**) averaging subcarrier fusion; (**b**) PCA subcarrier fusion.

Although denoised by the two-level filters, CSI is still redundant with six streams and thirty subcarriers in each stream. The multidimensional CSI data should first be fused to limit the redundancy without losing representative features before feature extraction. As shown in Figure 4, thirty CSI subcarriers in each CSI stream are highly correlated with each other. Moreover, Figure 5 illustrates the six CSI streams in one CSI dataset after subcarrier fusion using PCA and averaging, where individual streams vary independently from each other because CSI is collected from antenna pairs with distinctive spatial characteristics; this observation is also presented in [6].

Unlike existing CSI systems that eliminate bad streams to avoid miss-detection because of “bad streams” [35] or even simply average CSI successive subcarriers into one value [6], we propose a correlation-based fusion method that applies averaging to fuse highly correlated CSI subcarriers in each stream and PCA to fuse two most highly correlated CSI streams in each CSI dataset based on the above observation. PCA has been used as a statistical method in various applications, such as biomedical signals and computer graphics, and has also been introduced in CSI motion detection applications [36], which is a quantitatively rigorous method for reducing redundancy. PCA generates new variables called principal components. Each principal component is a linear combination of the original variables. All principal components are orthogonal to each other and form the orthogonal basis for the original data; thus, no redundant information exists. Therefore, PCA fusion properly extracts principal components from low-correlative redundant data. Averaging is a simple and efficient method to fuse high-correlative redundant data with low computational cost.

### 4.3. MVS Segmentation

After data preprocessing, CSI data are qualified as input of feature extraction, where the meaningful CSI stream caused by human motion should be initially segmented. To increase the segmentation accuracy, we present an MVS method to amplify the CSI stream fluctuation. The main concept is to compute the moving variance along the entire CSI stream step by step, in each step, a moving variance is calculated over a sliding window of length *L* across neighboring CSI packets, and the window is centered about the CSI packet in the current position. The high-level moving variance value denotes significant fluctuation of the CSI stream caused by dynamic human motion, whereas the low-level moving variance value denotes slight fluctuation of the CSI stream maintained by stable surroundings. Therefore, the CSI stream caused by motion can be segmented more accurately. For a CSI stream composed of *n* packets, the moving variance is defined as in Equation (6), where μ and *j* are the mean and packet number in the sliding window of length *L*, respectively, and *i* is the current position packet number in the entire CSI stream:

(6) $$\begin{array}{cc} CSI_{mv} & =\sum_{i=1}^{n}\left[\frac{1}{L-1}\sum_{j=1}^{L}{\left|CSI_{j\in L}-\mu \right|}^{2}\right],\\ \mu & =\frac{1}{L}\sum_{j=1}^{L}CSI_{j}. \end{array}$$

**Figure 6:**
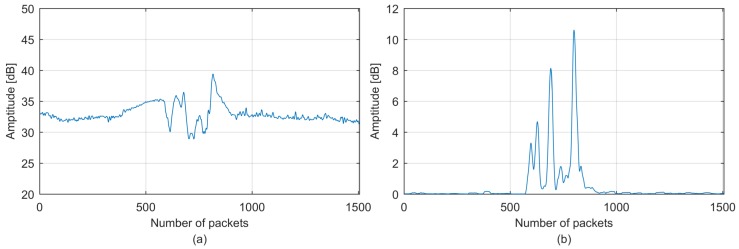
MVS segmentation: (**a**) the fused CSI stream; (**b**) corresponding moving variance sequence.

The CSI stream and corresponding moving variance stream of walking are shown in Figure 6, where each moving variance is calculated using a sliding window with an empirically selected length *L* of 30 across neighboring CSI packets. Note that a small *L* may result in an insignificant result and a large *L* may lead to a nonsensitive result. As expected, the moving variance stream is highly related to the CSI stream, and the meaningful fluctuation caused by motion becomes greater in magnitude and the non-meaningful fluctuation caused by temporal measurement variance (for example, the variance introduced by the experimental data recorder) becomes smaller. In particular, the start and end points of a significant CSI stream part caused by human motion are amplified by the start and end points of the fluctuation part of a moving variance stream. Therefore, the meaningful CSI stream part caused by human motion becomes more sensitive to segmentation to improve system performance.

### 4.4. Feature Extraction

CSI represents fine-grained data, and both the time and frequency domain features have the potential to form concrete feature profiles of candidate motions. First, six features in the time domain are adopted: (1) The normalized standard deviation (STD) is calculated to reflect the scale of dynamic motion, and dramatic scale motion always results in a large STD. (2) The median absolute deviation (MAD) is used as a robust measurement of the variability of a univariate sample of quantitative data. The sum of the squared MAD can express the concentration of data. (3) The interquartile range (IR) is also extracted to indicate the dispersion of the CSI variance. (4) The signal Shannon entropy (SSE) is a generic measure of system disorganization and is used to reflect the disorganization of CSI variance. (5) Peak-to-peak (PtP) indicates the difference between the maximum and minimum amplitudes of the received signal. (6) The time duration (TD) of each type of motion is unique and essential for classification accuracy.

**Figure 7:**
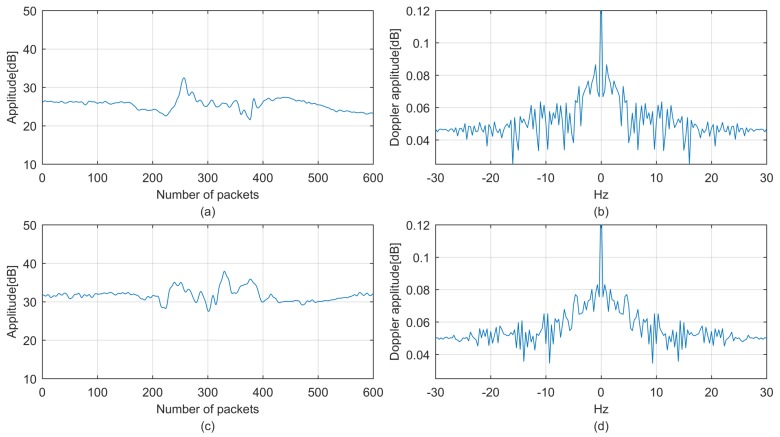
CSI profiles: (**a**) walking time profile; (**b**) walking Doppler frequency profile; (**c**) running time profile; (**d**) running Doppler frequency profile.

Aside from time domain features, frequency domain features also benefit motion detection accuracy. The Doppler spread spectrum is adopted in [3] to analyze on-body channel variances for wearable sensor-based motion detection for firefighter safety. It also has the potential to reflect the CSI stream temporal variation caused by human motions with different velocity and intensity such as walking and running. In our work, the Doppler spread spectrum is adopted to extract frequency domain features and derived from the Fourier transform of the time domain CSI data as shown in Equation (7), where $N_{p}$ is the number of CSI packets. Profiles of walking and running on flat floors are measured in the time and frequency domains as shown in Figure 7. As expected, more energy is distributed on the high-frequency components in the running profile than in the walking profile:

(7) $$\begin{array}{c} D\left(\lambda \right)=\sum_{i=1}^{N_{p}}csi_{i}e^{-j\frac{2\pi }{N}\lambda }. \end{array}$$

The Doppler spread spectrum is a symmetrical function with a zero mean and is always bounded by the maximum Doppler shift. An empirically selected threshold is adopted for each Doppler spectrum to obtain the final readings of the Doppler spread $S_{D(\lambda )}$. In addition, the root-mean-square Doppler spread (also known as the second central moment of the Doppler spectrum) is calculated as a frequency feature indicating the pattern of relative movement between the transmitter and receiver, given as

(8) $$R_{D(\lambda )}=\sqrt{\frac{\sum_{-f_{m}}^{f_{m}}\lambda^{2}D\left(\lambda \right)}{\sum_{-f_{m}}^{f_{m}}D\left(\lambda \right)}},$$

where $\pm f_{m}$ denotes the maximum Doppler shift, which is obtained from calculating D(λ) because it is always bounded by $\pm f_{m}$. In addition, we adopt the significant peak value as a motion feature, calculated as

(9) $$P_{D(\lambda )}=\max_{\lambda \in (-f_{m},f_{m})}D\left(\lambda \right).$$

The expectation value D(λ) is also employed as a reference for comparison with the real-time Doppler spread reading $S_{D(\lambda )}$ and is given by

(10) $$\mu_{D}=\frac{\sum_{-f_{m}}^{+f_{m}}D_{\left(\lambda \right)}}{\left|2f_{m}\right|}.$$

### 4.5. Correlation Check

Because highly correlated features have similar classification efficiencies, for all possible pair combinations of available features, we implemented a correlation check to limit the redundant features utilized in the classification. The correlation coefficient is used as the evaluation metric, and the formulation is given in Equation (11), where $v_{p}$ and $v_{q}$ represent different feature vectors. Notation $\bar{•}$ denotes the expectation of a feature vector, and $s_{v_{p}}$ and $s_{v_{q}}$ denote the standard deviations of the feature vector:

(11) $$\begin{array}{cc} r_{v_{p},v_{q}} & =\frac{\sum_{i=1}^{L}\left({\left(v_{p}\right)}_{i}-\bar{v_{p}}\right)\left({\left(v_{q}\right)}_{i}-\bar{v_{q}}\right)}{\left(L-1\right)s_{v_{p}}s_{v_{q}}},\\ p,q & \in \left\{1,10\right\},\\ p & \neq q. \end{array}$$

The calculated results are provided in Table 1, with only half of the matrix presented due to its symmetrical nature. The blue and red numbers are the intra-correlations of feature pairs in the time domain and frequency domain, respectively. The other areas provide the inter-correlations between time and frequency domain feature pairs. A correlation coefficient greater than 0.5 is considered to indicate inadequate efficiency, and these feature pairs are labeled in bold font. Several valuable observations can be made from the correlation check. First, for frequency domain features, $\mu_{D}$ is highly correlated with the others. Second, for time domain features, the STD, IR and MAD are strongly correlated, which can be implied from the fact that the STD and IR are proportional to the MAD according to the respective channel variations caused by human motions. Third, the inter-domain correlation is low, except for the correlations between entropy SSE and frequency features, which support the mechanism for feature extraction in previous sections to some extent. Such a correlation check provides the potential to optimize feature selection to reduce the redundancy of features utilized in classification, and the system performances of utilizing selected features will be discussed in Section 5.

## 5. Performance Comparison

### 5.1. Comparison Metrics

We adopt the confusion matrix as shown in Table 2 to evaluate the proposed system performance. The output of the individual classification case can be either target motion or other motion. The true classification rate is computed as the probability of correctly classifying a target sample. Correct sample classification is vital for indoor motion detection applications, such as detecting the abnormal motion of falling and detecting room intruders. The false rate indicates the probability of incorrectly classifying other samples in the total number of other samples. The true and false rates are expressed as shown in Equation (13). The ideal performance of a motion detection system includes a high true classification rate and a low false classification rate:

(12) $$\begin{array}{ccc} True\;rate & =\frac{TP}{TP+FP^{{}^{\prime }}};\;\;False\;rate & =\frac{FN}{FN+TN^{{}^{\prime }}}. \end{array}$$

In addition, precision, recall and F1 score are used to analyze results of the confusion matrix. Precision is the positive predictive value, recall is the sensitivity, and F1 score or the F-measure is the weighted average of both the precision and the recall. These three mathematics rules are as the following:

(13) $$\begin{array}{cc} Precision & =\frac{TP}{TP+FP^{{}^{\prime }}},\\ Recall & =\frac{TP}{TP+FN^{{}^{\prime }}},\\ F1=2\times & \frac{precision\times recall}{precision+{recall}^{\prime }}. \end{array}$$

### 5.2. Comparison Scheme

**Figure 8:**
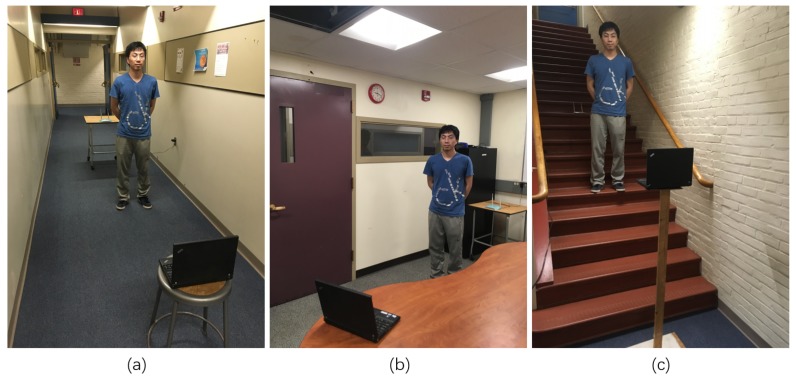
Experimental environments: (**a**) corridor; (**b**) room; (**c**) staircase.

In our experiments, the Wi-Fi transmitter is a common commercial Wi-Fi router with two antennas, the receiver is a laptop equipped with an Intel 5300 NIC, and the Wi-Fi transmitter and receiver are always located at the same horizontal level. The CSI collection software is the open source CSI tool presented by Halperin et al., and the Intel 5300 NIC reports CSI for thirty subcarriers spread evenly among the 56 subcarriers of a 20-MHz channel or the 114 carriers in a 40-MHz channel [33]. We conduct experiments at the Atwater Kent Laboratories building of the Worcester Polytechnic Institute with eight student volunteers (four males and four females). The volunteers repeat each candidate motion in a real indoor environment setting, such as a corridor, room or staircase. The candidate motion set is consistent with the most frequent indoor motions, namely, falling, running, walking and sitting. Because motion in different locations generates different multi-path and shadow fading effects, the volunteers always perform motions in the middle of the Wi-Fi transmitter and receiver, and the distance between the transmitter and receiver is approximately 5 m. The candidate motions are illustrated in Table 3 and the experiment setting is shown in Figure 8.

**Figure 9:**
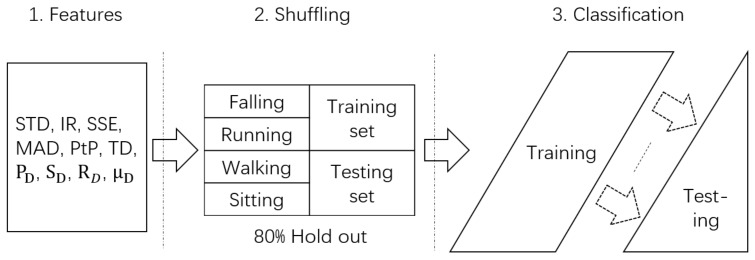
Classification data flow.

In the training set, each specific motion lasts for approximately 3 s, and the sample number obtained for each type of motion is 50 (samples) * 8 (volunteers) = 400, where each sample is segmented from one CSI stream consisting of 1600 CSI packets. In addition, four types of motions are performed in two flat floor environments (i.e., a corridor and a room), with 400 (samples/each type of motion) * 4 (types of motions) * 2 (environments) = 3200 samples. Four types of motion are performed in only one staircase environment, and the sample number is 400 (samples/each type of motion) * 4 (types of motions) * 1 (environment) = 1600. Subsequently, empirical samples are forwarded into the shuffling phase, where the entire dataset of 4800 samples is randomly shuffled to avoid any possible sequential effects. The shuffling process is necessary because involving randomness helps mitigate the effect of human error, such as various subject body sizes and habitual differences in subject posture [3]. Twenty percent of the samples are reserved as a testing set for future performance validation, and the remaining 80 percent of the samples are used to train the classifier. The overall data flow of the proposed classification scheme is shown in Figure 9.

For classification, machine learning RF is employed to achieve multiple candidate classifications. RF is a machine learning algorithm that integrates multiple trees via integrated learning. The multiple decision trees with an empirically selected amount of 150 are trained as the elementary elements and further integrated as RF in our work. In addition, because certain features are correlated with others, a stepwise correlation check is conducted based on the correlation check result introduced in Section 4 to reduce the redundancy of features utilized in the classification. The main concept is to evaluate the combined effect of candidate features in classification by removing one feature at a time.

### 5.3. Comparison of Data Fusion Methods

**Figure 10:**
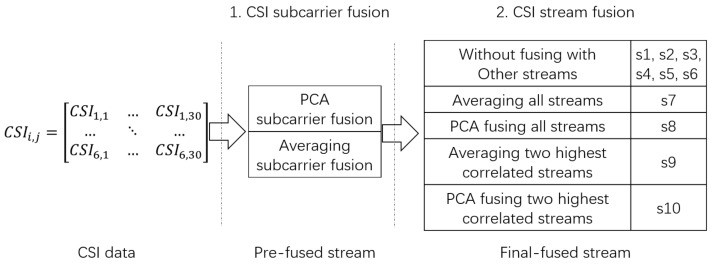
CSI subcarrier and stream data fusion.

To verify our proposed correlation-based CSI fusion method and explore the details of how different fusion methods affect classification, candidate motions are classified with different combinations of PCA and averaging fusion. As described in Section 3, each entire CSI dataset consists of six streams and thirty subcarriers in each stream. For one CSI dataset, we first apply PCA and averaging fusion to thirty subcarriers in each stream separately and categorize the pre-fused streams as: (1) the PCA subcarrier fused stream and (2) the averaging subcarrier fused stream. Then, we further apply the two fusion methods to selected pre-fused streams based on the correlations within them. The final-fused streams are categorized as: (1) the 1st, 2nd, 3rd, 4th, 5th, and 6th streams without fusion with others are denoted by s1, s2, s3, s4, s5, and s6, respectively; (2) the stream generated by averaging all streams is denoted by s7; (3) the stream generated by PCA fusion of all streams is denoted by s8; (4) the stream generated by averaging the two most highly correlated streams is denoted by s9; and (5) the stream generated by PCA fusion of the two most highly correlated streams is denoted by s10. The data fusion flow chart is shown in Figure 10.

**Figure 11:**
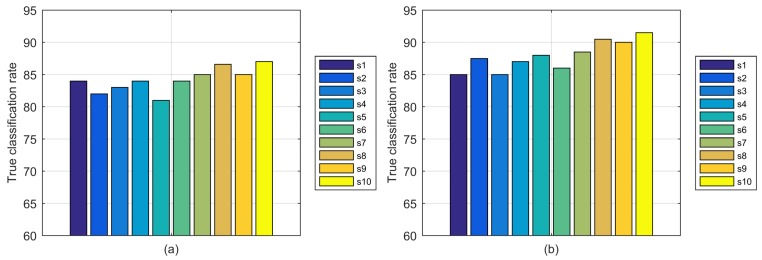
Classification results using different data fusion methods: (**a**) PCA subcarrier fusion; (**b**) averaging subcarrier fusion.

The classification results using different data fusion methods are shown in Figure 11. First, the average precision based on averaging subcarrier fusion is 87.9%, which is 4% higher than 84.2% obtained with PCA subcarrier fusion. Because the subcarrier in each stream is highly correlated as discussed in Section 4, the averaging computation has better performance in aggregating highly correlative CSI subcarrier data compared to PCA, which extracts representative components but also loses certain features. Second, based on averaging subcarrier fusion, the average precision over the final-fused stream from s7 to s10 is 90.1% versus 86.4% for final-fused streams s1 to s6, confirming that stream fusion is beneficial for CSI motion detection. The highest precision is obtained over final-fused stream s10 using either PCA or averaging subcarrier fusion, which is generated by PCA stream fusion over the two most highly correlated pre-fused streams. Therefore, the experiments validate that averaging fusion is appropriate for highly correlated subcarriers, and PCA fusion is appropriate for streams, further verifying the efficiency of our proposed correlation-based data fusion method.

### 5.4. Comparison of Utilizing Selected Features

To limit the redundancy of features utilized in classification, we evaluate the combined effects of available features by removing one feature at one time based on the correlation check result in Section 4 in the order of MAD, $\mu_{D}$, TD, $S_{D}$, $P_{D}$, $R_{D}$ and STD. Because the average true classification rates for all candidate motions approach baseline, the remaining features of the above-mentioned removal process are considered a minimum set for effective classification. Candidate motions in flat floor environments are classified, and average true classification rates are recorded. We repeat both the training and testing processes each time a feature is removed. The remaining feature combination and respective classification rate are provided in Table 4.

As expected, fewer features result in lower accuracy in general. However, two features, namely, MAD and $\mu_{D}$, influence the accuracy only marginally. Therefore, an acceptable accuracy can still be achieved with only eight other important features. In addition, the time feature TD improves the accuracy significantly from 68.4% to 89.8%, which verifies that the time duration of motion as a candidate feature has an important impact on classification. In the following sections, all experiments are conducted using only the subset of available features to reduce the computational cost.

### 5.5. Comparison of Fall Detection Performance

Injuries caused by falls have been regarded as one of the major health threats among the elderly. Therefore, several recent studies have focused on CSI-based fall detection; however, they are mainly designed for flat floor environments and disregard complex indoor scenarios, such as a staircase. We implemented our system to detect falling and distinguished it from the most frequent indoor motions, including walking, running and sitting, in both flat floor and staircase environments, including rooms, corridors and staircases.

**Figure 12:**
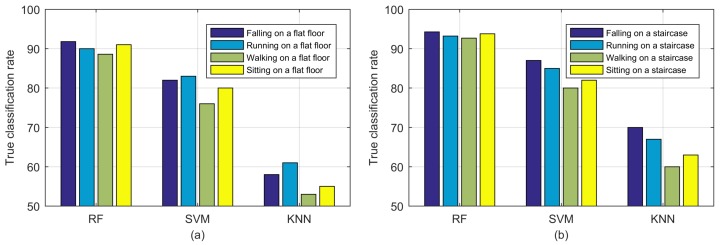
Fall detection results: (**a**) flat floor environment; (**b**) staircase environment.

We first present the confusion matrix of the experimental results in Table 5. The accuracy is always higher on the staircase than on the flat floor. The accuracy of falling is 94% on a staircase, which is higher than that on the flat floor (92%). For running, walking and sitting, the accuracy on a staircase is 93%, 93% and 94% versus 90%, 89%, and 91% on a flat floor, respectively. In addition, the precision, recall and F1 score are higher in staircase environments with 95%, 94% and 94.5% versus 92%, 89% and 90.5% in flat floor environments and also validate the better performance in staircase environments. Second, the SVM and KNN algorithms have been applied in experiments to validate the efficiency of the RF algorithm employed in our proposed classification scheme. SVM uses kernel functions to solve the randomness and incompleteness of CSI values [13], and in the landmark work by Yishuang Geng et al. [3], SVM has been selected as the primary machine learning algorithm for first responder wearable sensor based motion classification and achieved satisfactory accuracy; KNN has also been introduced as a nonparametric method for fall detection [37]. The classification results for different algorithms are illustrated in Figure 12. The motion detection accuracy using the RF algorithm is always higher than that using the SVM and KNN algorithms, and the fall detection accuracy is higher on staircases than that on flat floors regardless of the algorithm used. In addition, compared with the CSI fall detection system WiFall (89% in a flat floor environment, a dormitory), our system improves the fall detection accuracy to 92% and can also detect falling on a staircase with a higher accuracy of 94%.

In conclusion, our system can detect falling in both flat floor and staircase indoor environments in complicated indoor scenarios, such as a hospital with elderly individuals or patients. Moreover, our experiments also verify the potential and better performance of CSI-based fall detection on a staircase versus a flat floor as in the previous analysis in Section 3.

### 5.6. Comparison of Intruder Detection Performance

Aside from human health care, CSI motion detection systems have also been applied to detect intruders by distinguishing human gaits for human security as individuals have unique motion characteristics, such as action habits and intensities. For example, young people always walk faster and more powerfully than the elderly, and the CSI variance generated by them is distinctive and can be applied to identify family members or detect intruders. In experiments to evaluate our system ability to detect intruders, we label one of the eight experimental volunteers as an intruder and distinguish the intruder from other volunteers using human walking and running gait CSI features in both flat floor and staircase environments.

**Figure 13:**
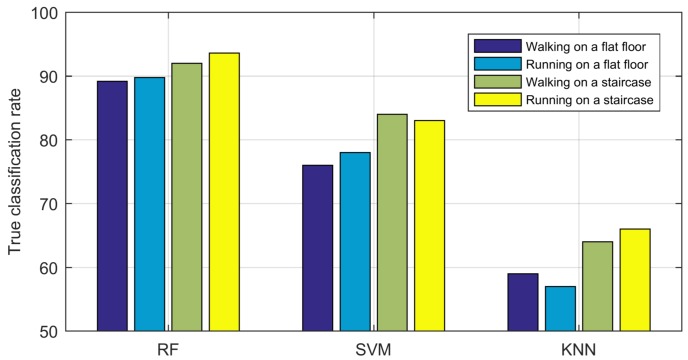
Intruder detection results using different algorithms.

As shown in Figure 13, similar to the fall detection experiments, the intruder detection accuracy are higher on a staircase than on a flat floor regardless of the classification algorithm used. The algorithms can be ranked in terms of highest average accuracy as RF, SVM and KNN, also validating the efficiency of our proposed RF algorithm in the CSI motion detection system. For intruder detection using the RF algorithm, the accuracy using walking and running gait features are 92% and 93.6% on a staircase versus 89.2% and 90% on a flat floor, respectively. Moreover, compared with the gait-based CSI intruder detection system CareFi (the average accuracy is 87% with one pair of access point and monitor point), our system detects an intruder with higher average accuracy on a flat floor at 90% and can also be used on a staircase with a better average accuracy of 93%.

## 6. Conclusions

In this work, we proposed a novel indoor CSI motion detection system and validated the potential and better performance of CSI motion detection in staircase environments versus flat floor environments through experiments of fall detection and intruder detection. In addition, we presented several novel methods for CSI motion detection including correlation-based data fusion, MVS segmentation method, Doppler spread spectrum to improve the system performance, and a stepwise correlation check to reduce the implementation cost.

## Figures and Tables

**Table 1:** Correlation check of available features.

	STD	IR	SSE	MAD	PtP	TD	$P_{D}$	$S_{D}$	$R_{D}$	$\mu_{D}$
**STD**	1	**0.873**	0.263	**0.981**	**0.872**	0.189	0.293	0.114	0.264	0.254
**IR**		1	0.325	**0.926**	**0.635**	0.189	0.364	0.072	0.315	0.298
**SSE**			1	0.294	0.067	0.141	**0.996**	0.336	**0.607**	**0.885**
**MAD**				1	**0.797**	0.199	0.328	0.110	0.295	0.273
**PtP**					1	0.191	0.086	0.136	0.127	0.095
**TD**						1	0.143	0.093	0.058	0.143
$P_{D}$							1	0.309	**0.588**	**0.869**
$S_{D}$								1	0.237	0.429
$R_{D}$									1	**0.752**
$\mu_{D}$										1

**Table 2:** Confusion matrix.

	Predicted Target	Predicted Other
Actual Target	True Positive (TP)	False Negative (FN)
Actual Other	False Positive (FP)	True Negative (TN)

**Table 3:** Candidate motions in the two environments.

Scenarios	Flat Floor		Staircase
	Room	Corridor	
Candidate motions	Walking	Walking	Walking
	Sitting	Sitting	Sitting
	Running	Running	Running
	Falling	Falling	Falling

**Table 4:** Classification by taking away one feature at one time.

Number of Features	Combinations of Selected Features										Classification Rate (%)
	MAD	IR	SSE	STD	PtP	TD	$P_{D}$	$S_{D}$	$R_{D}$	$\mu_{D}$	
3		×	×		×						25.3%
4		×	×	×	×						32.9%
5		×	×	×	×				×		40.2%
6		×	×	×	×		×		×		56.7%
7		×	×	×	×		×	×	×		68.4%
8		×	×	×	×	×	×	×	×		89.8%
9		×	×	×	×	×	×	×	×	×	90.4%
10	×	×	×	×	×	×	×	×	×	×	90.6%

**Table 5:** Confusion matrix of fall detection results.

Flat Floor		Actual			
Environment		Falling	Running	Walking	Sitting
Predicted	Falling	92%	1%	2%	5%
	Running	1%	90%	6%	2%
	Walking	3%	7%	89%	1%
	Sitting	7%	0%	2%	91%
**Staircase**		**Actual**			
**Environment**		**Falling**	**Running**	**Walking**	**Sitting**
Predicted	Falling	94%	0%	2%	3%
	Running	1%	93%	6%	0%
	Walking	0%	4%	93%	3%
	Sitting	5%	0%	1%	94%
